# Sustainable Surface Modification of Polyetheretherketone (PEEK) Implants by Hydroxyapatite/Silica Coating—An In Vivo Animal Study

**DOI:** 10.3390/ma14164589

**Published:** 2021-08-16

**Authors:** Thomas Frankenberger, Constantin Leon Graw, Nadja Engel, Thomas Gerber, Bernhard Frerich, Michael Dau

**Affiliations:** 1 Institute of Physics, Rostock University, 18057 Rostock, Germany; thomas.frankenberger@uni-rostock.de (T.F.); thomas.gerber@uni-rostock.de (T.G.); 2 Department of Oral, Maxillofacial and Plastic Surgery, Rostock University Medical Center, 18057 Rostock, Germany; constantin.graw@uni-rostock.de (C.L.G.); nadja.engel@med.uni-rostock.de (N.E.); bernhard.frerich@med.uni-rostock.de (B.F.)

**Keywords:** polyetheretherketone (PEEK) implants, silicon dioxide, hydroxyapatite (HA), animal rat model, mechanical testing, tissue integration, implant interface, osteoconductive modification, pull-out

## Abstract

Polyetheretherketone (PEEK) has the potential to overcome some of the disadvantages of titanium interbody implants in anterior cervical and discectomy and fusion (ACDF). However, PEEK shows an inferior biological behavior regarding osseointegration and bioactivity. Therefore, the aim of the study was to create a bioactive surface coating on PEEK implants with a unique nanopore structure enabling the generation of a long-lasting interfacial composite layer between coating material and implant. Seventy-two PEEK implants—each thirty-six pure PEEK implants (PI) and thirty-six PEEK implants with a sprayed coating consisting of nanocrystalline hydroxyapatite (ncHA) embedded in a silica matrix and interfacial composite layer (SPI)—were inserted in the femoral condyles of adult rats using a split-side model. After 2, 4 and 8 weeks, the femur bones were harvested. Half of the femur bones were used in histological and histomorphometrical analyses. Additionally, pull-out tests were performed in the second half. Postoperative healing was uneventful for all animals, and no postoperative complications were observed. Considerable crestal and medullary bone remodeling could be found around all implants, with faster bone formation around the SPI and fewer regions with fibrous tissue barriers between implant and bone. Histomorphometrical analyses showed a higher bone to implant contact (BIC) in SPI after 4 and 8 weeks (*p* < 0.05). Pull-out tests revealed higher pull-out forces in SPI at all time points (*p* < 0.01). The presented findings demonstrate that a combination of a bioactive coating and the permanent chemical and structural modified interfacial composite layer can improve bone formation at the implant surface by creating a sustainable bone-implant interface. This might be a promising way to overcome the bioinert surface property of PEEK-based implants.

## 1. Introduction

Interbody cage implants filled with bone graft substitutes are one of the main methods in anterior cervical discectomy and fusion (ACDF) and a safe, as well as efficient, alternative to iliac crest bone autografts [1]. These implants are mainly made of titanium (Ti), polyetheretherketone (PEEK) and carbon fiber PEEK. While Ti supports the osseointegration of the implants [2], PEEK offers the advantage of radiolucency and an elastic modulus closer to the bone, resulting in theoretically reduced levels of subsidence [3]. However, the bioinert property of PEEK limits the interaction with surrounding tissue, making PEEK inferior to titanium as an implant material [4]. Numerous animal studies have histologically and histomorphometrically proven that this limitation leads to a fibrous tissue barrier between the PEEK implant and newly formed bone, resulting in poor osseointegration [5].

Modifying the surface properties of PEEK implants might be a way to increase osseointegration. Therefore, various modifications for improving the biological behavior regarding osseointegration were tested [6,7,8,9,10,11,12,13,14,15].

The typical hydrophobic surface can be turned into more hydrophilic by a combination of immersions with H_2_SO_4_ and NaOH. This ambient temperature sulfonation, which reduced the PEEK water contact angle from 78 to 37 degrees, seems to be more effective than conventional plasma treatments [9]. Another technique to change the surface is by using a treatment with accelerated neutral atom beam (ANAB) technology. The human osteoblast-like cells seeded on the modified surface showed enhanced proliferation and increased expression of ALPL (alkaline phosphatase-like proteins), RUNX2 (runt-related transcription factor 2), COL1A (collagen type I alpha), IBSP (integrin-binding sialoprotein) and BMP2 (bone morphogenetic protein 2) [6]. These effects on the cellular level were connected with an observed increased bone contact and push-out force in a sheep animal model [16].

One of the key factors for biological integration is the early adsorption of proteins and cell colonization at the surface of the implants. Improving cell adhesion can be achieved by modifying the implant surface [8]. Typically, PEEK implants have a smooth surface, which is generally known to limit osseointegration [17]. One simple approach to overcome this disadvantage of PEEK implants is roughening the surface [11]. Micro roughened PEEK implant surfaces showed a higher cell proliferation accompanied by an increased osteocalcin (OC) expression and bone-like nodule formation on the surface [17], as well as higher pull-out force compared to mirror-polished PEEK [17]. Whereas introducing nano roughness to the PEEK surface promotes especially the adsorption of growth factors and proteins, which are known to play a crucial role in the initial cellular mechanisms of osseointegration [18,19].

Another approach to turn the bioinert PEEK surface into a more bioactive one is the surface coating with biomaterials such as alumina [12], calcium phosphate (CaP) [14], especially hydroxyapatite (HA) [13]. Materials such as HA have good osteoconductive properties [20] due to their similar composition to the mineralized part of the bone. HA applied as a coating to implant surfaces was found to increase the rate of bone formation around the implant and enhance the osseointegration [21]. While there is a whole range of different HA materials, nanocrystalline hydroxyapatite is known to deliver good clinical results [22]. Regardless of the good biological behavior of HA applied as a coating, mechanical ablation during the implantation process and delamination of the coating results only in a time-limited improvement of the implant surface properties regarding bioactivity. In order to utilize the bioactive properties of HA coating and to ensure a stable long-term osteointegration, the PEEK surface itself needs to be modified as well.

Achieving an adequately adhered coating on PEEK is a challenging task on its own and a necessary requirement for the success of the medical application. The chemical structure of PEEK limits the bonding strengths of bioactive coatings, e.g., HA, because of the absent chemical bond between the materials. One way to create a bonding is surface modifications of PEEK, which lead to functionalization of the repeat unit and establish dipole–dipole interactions with polar groups of the coating material [23]. Another point of view to achieve a strong bond is a firmly anchored coating on the substrate by creating interphase of both materials. Especially porous bioactive materials are well suited for melt infiltration of polymers in order to form a composite. Such a composite as an intermediate layer between a porous coating material and bulk PEEK provides a structural interlock and may prevent the delamination of the coating material.

Following our previous research on the bioactive coating of titan implants [24], the purpose of the study at hand was to histologically and histomorphometrically investigate the osseointegration of PEEK implants coated with a nanostructured SiO_2_/HA biomaterial with an intermediate composite layer as interface. We hypothesized that (i) the interfacial composite layer between the implant and the bioactive coating might enhance the bioactivity of PEEK and (ii) provide a long-term modification of the PEEK surface due to the increased bone conductive properties of the composite layer.

## 2. Materials and Methods

### 2.1. Study Materials

**Figure 1:**
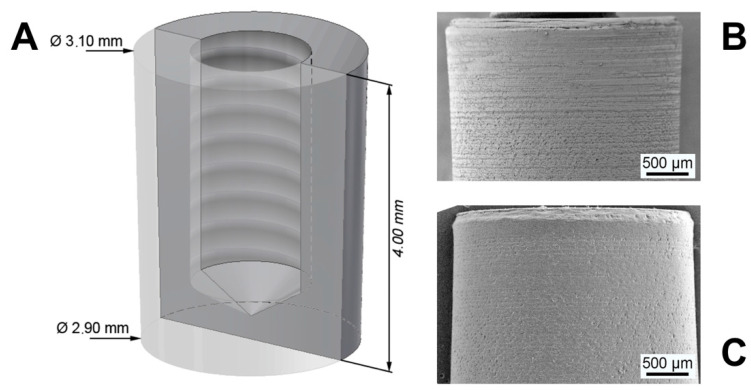
(**A**) Dimensions of PEEK implants. (**B**) SEM images of SiO_2_/HA-coated PEEK implant. (**C**) SEM images of pure PEEK implant.

The used custom frustum-like shape PEEK implants were made of machined medical grade PEEK (VESTAKEEP i4, Evonik Degussa GmbH, Essen, Germany) with a length of 4.00 mm and a diameter of 3.10 mm at the top and 2.90 mm at the bottom (Figure 1A). The frustum shape was chosen to fulfill the needs of the planed shear force-free pull-out test within the study. All implants were ultrasonically cleaned in deionized alkaline water and afterward generously rinsed with ethanol. Half of the implants (n = 36) were coated with the biomaterial (SPI). The remaining implants (n = 36) were left uncoated (PI). The coating was based upon the principle of the sol gel technique creating a highly porous silica matrix with embedded nanocrystalline hydroxyapatite as platelets with a length of 50–70 nm, 20–25 width and 3–4 nm thickness [24]. The silica sol, derived by acidic hydrolysis of tetraethyl orthosilicate (Sigma Aldrich, St. Louis, MO, USA) and HA (Artoss GmbH, Rostock, Germany), were dispersed in pure ethanol with a ratio HA:SiO_2_ of 61:39 wt.%. The dispersion was spin-spray-coated onto the implant surface and subsequently dried under dry oil-free airflow; see [5,24] for a thorough description of the complete process. After coating, the implants were heat-treated with a hot air stream above the melting temperature of PEEK in order to form an interface between the polymer and the coating material. During the heat treatment at 400 °C, the implant surface was locally molten in the range of viscous flow behavior leading to the melt infiltration of the polymer into the unique interconnected pore structure of the coating material. Both groups, coated and uncoated implants, were sterilized in a vacuum oven at 160 °C for 24 h [25].

### 2.2. Material Characterization

Scanning electron microscopy (Merlin VP compact, Zeiss, Germany) analyses were performed on uncoated and coated implants before (Figure 1B,C) and after the in vivo experiments. In order to render the surface conductive, all implants were sputtered with a Cu layer of approximately 5 nm. Images were taken at different magnifications with an acceleration voltage of 10 keV. The local chemical composition was determined by energy-dispersive X-ray analysis (EDX).

The nanostructure of cross-sectional slices was investigated by transmission electron microscopy (EM912, Zeiss, Germany) with an acceleration voltage of 120 keV. Samples were cut into thin sections with a thickness of about 20 to 50 nm by using an ultramicrotome (Leica EM UC6, Leica, Germany) and subsequently were placed on copper grids.

### 2.3. Animal Model and Procedures

A well-established animal rat model was used in the study at hand [26,27]. All animal experiments were approved by the State Office of Agriculture, Food Safety and Fishery Mecklenburg-Vorpommern, Germany (LALLF M-V/TSD/7221.3-1-040/18) and followed the National Institutes of Health guidelines for the care and use of laboratory animals, as well the ARRIVE Guidelines for Reporting Animal Research [28].

**Figure 2:**
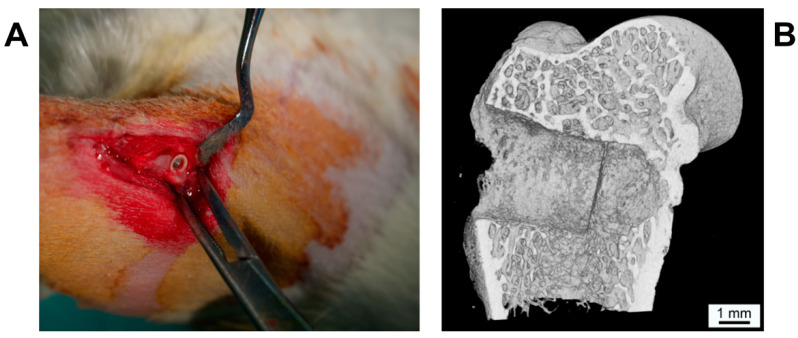
(**A**) Intraoperative picture of inserted implant in lateral aspect of femur. (**B**) µCT image of the bony defect in lateral femur condyle with SiO_2_/HA-coated PEEK implant (SPI), 4 weeks postoperative. The PEEK implant is radiolucent and thus not visible.

Thirty-six male adult Wistar rats with an average mean body weight of 578.4 g ± 55.6 g were included in the study. The animals were kept at the laboratory with dry food and water ad libitum under standard conditions, including an artificial light–dark cycle of 12 h. All performed surgical interventions were made under general anesthesia with isoflurane (Forene^®^, AbbVie Germany GmbH and Co. KG, Ludwigshafen, Germany) and intramuscular analgesia via 0.5 mg carprofenum (Rimadyl^®^, Zoetis Germany GmbH, Berlin, Germany). Following anesthesia, the lateral epicondylus of the femur was exposed via longitudinal incisions at the lateral aspect of the femur. After drilling the implant hole (diameter 3.0 mm, Figure 2) using a sodium chloride cooled implant burr (pilot drill bur G1001 RAXL, Hager and Meisinger GmbH, Neuss, Germany), the implants—randomly chosen by using a computer-generated list—were inserted. In each animal, one implant was inserted on each side, resulting in a total of seventy-two implants (SPI: n = 36; PI: n = 36). After closing periosteum, muscle and skin in layers using absorbable suture material (Vicryl^®^, Ethicon, Norderstedt, Germany), the animals received intramuscular analgesia via 0.5 mg carprofenum (Rimadyl^®^, Zoetis Germany GmbH, Berlin, Germany). Additionally, metamizol natrium (Novaminsulfon-ratiopharm^®^, ratio-pharm GmbH, Ulm, Germany) was applied in the drinking water on a daily basis for the first 5 days postoperatively.

The animals were sacrificed after 2, 4 and 8 weeks (24 implants each time point) by carbon dioxide gas inhalation and bleeding dry. The femurs were harvested for further histological examination (n = 36) and pull-out test (n = 36).

### 2.4. Pull-Out Tests

After cleaning the harvested femurs from residual soft tissue, the distal portion of the distal part of the femora was separated. The gathered bone samples with the implants were wrapped in gauze soaked with saline and stored at −40 °C between 4 and 8 weeks. Before performing the pull-out tests, the samples were thawed at 6 °C overnight in saline solution for the sake of rehydration. Subsequently, the samples were prepared for the pull-out analyses by embedding in a polyurethane-based resin (RenCast FC 52/53, FC 52 Polyol with a ratio of 50:50 wt.%, Huntsman, Belgium) for fixation in a custom-made tool to align the implant parallel to the vector of the applied force. After curing and alignment, implants were pulled out with a displacement speed of 0.5 mm/min using a universal testing machine (BT1-FR1.0Tn.140, Zwick GmbH and Co. KG, Pforzheim, Germany) equipped with a 200 N measuring box. The maximal load, which is defined as the load of implant loosening, was recorded for n = 6 implants of each group and time point.

### 2.5. Histological Procedures and Histomorphometrical Measurement

Following fixation in 4% phosphate-buffered formaldehyde for two weeks, the femur bone was dehydrated with alcohol in ascending order (70%–80%–90%–96%–100% each for 24 h and 100% for 48 h) for 7 days. Afterward, xylene was used twice (for 24 h and for 48 h). The specimens were embedded in combination (Technovit 9100^®^ VLC; Heraeus Kulzer, Hanau, Germany) of methyl methacrylate (MMA) and ultraviolet light-activated polymethyl methacrylate (PMMA) in sequential steps (stabilized MMA + Xylol for 48 h, stabilized MMA for 72 h, destabilized MMA for 72 h and destabilized MMA + PMMA). The slices were carefully photopolymerized and processed, applying the sawing and grinding technique [29] using a micro-grinding system (Exact, Norderstedt, Germany). The specimens were ground to a final thickness of 30–40 µm and stained with Giemsa and toluidine blue.

Histological and histomorphometrical analyses were performed for n = 6 implants of each group and time point in the Giemsa toluidine blue-stained sections using a light microscope (Zeiss Axioskop 40, Oberkochen, Germany). The bone to implant contact (BIC) along both sides of the implant was measured with the imaging software (Adobe Photoshop CS^®^ V12.0, Adobe Systems Software Ireland Ltd., Dublin, Ireland).

### 2.6. Statistics

All statistical analyses were performed with SPSS statistical package version 20.0 (SPSS Inc., Chicago, IL, USA). The results in the study at hand were expressed as arithmetic means ± standard deviation (SD). Before testing for statistical significances, all variables were evaluated for normal distribution via Shapiro–Wilk test. Depending on the presence or the absence of normal distribution, additional analyses were conducted using Mann–Whitney and Student’s t-test, respectively. *p*-values < 0.05 were considered as significant.

## 3. Results

### 3.1. Coating Characterization

**Figure 3:**
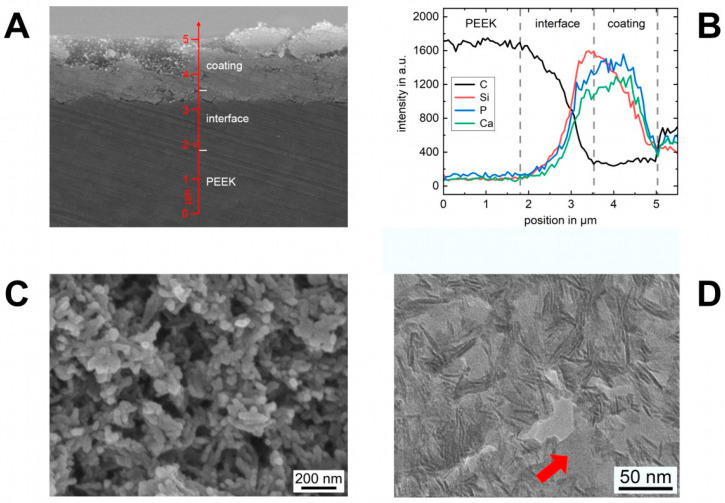
(**A**) SEM image of the cross-section of a coated and heat-treated implant. Three distinct areas are distinguishable: bulk PEEK, interface and coating material. (**B**) Chemical composition of the elements carbon, silicium, phosphor and calcium along the arrow in the SEM image (**A**). (**C**) SEM image of the nanostructure of the coating material with interconnecting pores. (**D**) TEM image of the interface cross-section. All pores of the coating material (darker contrast) are filled with PEEK (brighter contrast).

The coating process was successfully applied to the PEEK implants. Before coating, the bare PEEK implants offered a smooth surface with irregularities due to the machining process, see Figure 3A. These features were balanced by the coating material, which has fully covered the surface with a uniform layer. The thickness of the as-deposited coating was approximately 4–5 µm. An SEM image with high magnification in Figure 3C reveals the nanostructure of the coating material. HA nanocrystals are embedded in a highly porous silica matrix and form together bigger aggregates, creating pores in a size range of a few nanometers up to several hundred. Based on the wet-chemistry route, all pores are interconnected and generate a high surface of 146 m^2^/g with a mean pore size of 26.9 nm. The topography of the coating is characterized by open pores due to the morphology of the HA crystals and the silica matrix.

### 3.2. Interface Characterization

In Figure 3A, the cross-section of a coated implant with an interface is shown in an SEM image. The as-deposited coating material can be seen as a bright layer with a high electron intensity on top of the PEEK implant with less intensity. Locally molten PEEK from the implant surface partially infiltrated into the pore structure of the as-deposited coating material and filled the free pore volume. In the interface region, a dense composite layer arose which secondary electron intensity cannot be distinguished from the pure bulk PEEK. An analysis of the chemical composition perpendicular to the surface (Figure 3B) indicates at the beginning only a carbon signal belonging to bulk PEEK. As soon as the intensity of silicon, phosphorous and calcium rise, the interface starts as an overlap region of the elements occurring in the coating material (Si, P and Ca) and PEEK (C). Further, towards the top, the carbon signal drops and reaches a minimum, whereas the elements of the coating material follow an inverse behavior. Therefore, PEEK penetrated not the whole coating thickness. On top of the interface exists a residual portion of the coating material, which was not altered by melt infiltration, thus being unmodified. According to the SEM investigation, the interface has characteristics of a uniform layer with a thickness of approximately 2 µm and connects the bulk PEEK of the implant and the residual coating material as an intermediate layer.

The nanostructure of the interface is shown in the TEM image in Figure 3D. HA crystals, which are orientated parallel to the electron beam, were visible as acicular lines. The silica matrix around the HA crystals is not visible due to a similar scattering absorption contrast to PEEK. Together with the randomized HA crystals within the coating, the nanostructure of the coating material is specifiable through differences in the contrast. As can be seen, regardless of the size, all pores within the coating material were completely filled with PEEK, indicated as structureless areas with a brighter contrast (arrow).

### 3.3. In Vivo Experiments

For all animals, postoperative healing was uneventful, and no postoperative complications were observed.

### 3.4. Pull-Out Tests

**Figure 4:**
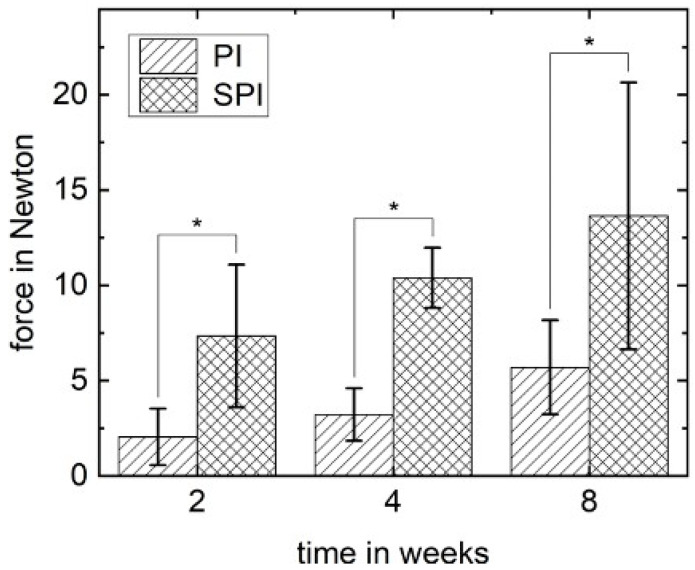
Results of the pull-out test for PI and SPI groups. The pull-out force (mean values in Newton) is pictured with standard deviation. The mean values for SPI were significantly higher for each time point (* *p* < 0.01).

The conducted pull-out tests ran smoothly without any complications. Analyses of the mean applied forces revealed an increase over time in both groups PI and SPI. The mean applied pull-out forces in SPI were about twice as high in PI at each point in time (2 weeks: 7.3 ± 3.7 versus 2.1 ± 1.5 N; 4 weeks: 10.4 ± 1.6 versus 3.2 ± 1.4 N; 8 weeks: 13.6 ± 7.0 versus 5.7 ± 2.5 N) and significantly increased compared to the PI at the same time points (*p* < 0.01). The results of the pull-out test are illustrated in Figure 4.

### 3.5. Histomorphometrical Analysis

**Figure 5:**
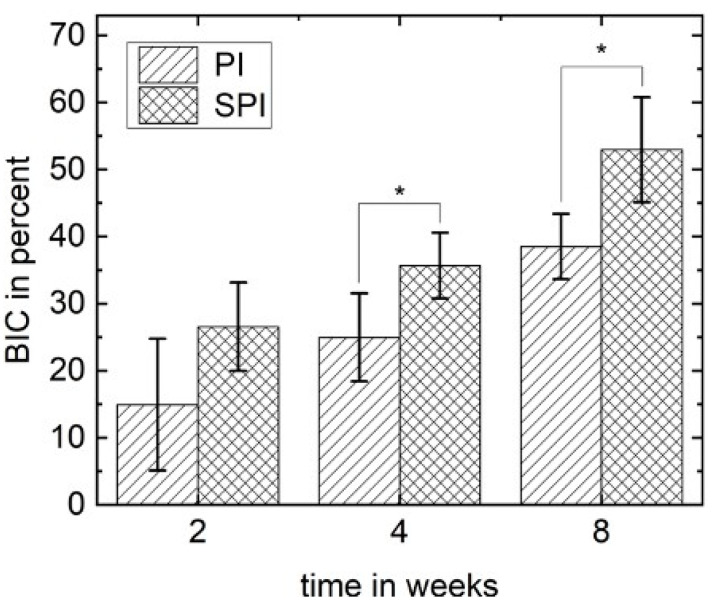
Bone to implant contact (BIC) of the PI and SPI group with standard deviation. The mean BIC of both groups increases as a function of time. After 4 and 8 weeks, the BIC is significantly higher for SPI (* *p* < 0.05).

Histomorphometrical analysis focusing on the bone to implant contact (BIC) showed a significant increase of the mean values over time in both groups, PI and SPI (*p* < 0.05). The BIC of SPI and PI after 2 weeks were not statistically significant (26.6 ± 6.6% versus 15.0 ± 9.8%). With increasing healing time, the BIC of the SPI was significantly higher than the corresponding value of the PI after 4 weeks (35.7 ± 4.9% versus 25.0 ± 6.6%) and 8 weeks (53.0 ± 7.8% versus 38.5 ± 4.9%). The results of the histomorphometrical analyses are illustrated in Figure 5.

### 3.6. Histological Analysis

**Figure 6:**
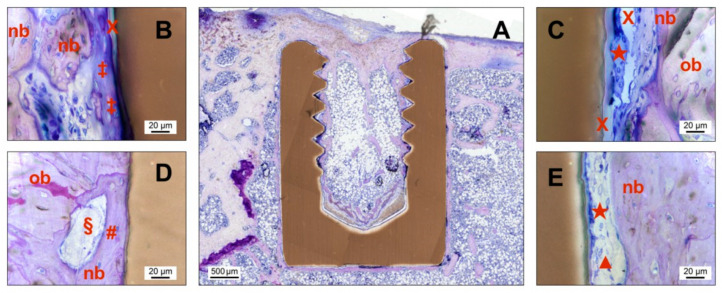
Images of undecalcified cut and ground sections of the PI and SPI group after different time points postoperatively. The sections were histologically stained with a mixture of Giemsa and toluidine blue. (**A**) Overview image of an uncoated pure PEEK implant (PI) after 8 weeks showing new bone formation around the implant outline. (**B**–**E**) Additional detail images with higher magnification focusing on the direct bone-implant contact area for SPI (2 weeks (**B**) and 8 weeks (**D**)) and PI (2 weeks (**C**) and 8 weeks (**E**)). Note: nb—new bone, ob—old bone, X—preparation artifact, ★—elongated irregular-shaped giant cell, ‡—osteoid, #—osteocyte in contact to the implant surface, §—blood vessel, ▲—fibroblast.

All implants of the PI and SPI groups showed osseointegration without any sign of inflammation or encapsulation, e.g., Figure 6A. Due to the frustum-like shape of the implants, the press-fit was established only in the cortical portion. Different amounts of newly formed bone were visible around the implants and in the residual drilling channel. Generally, bone formation continued over time, and implants were increasingly enframed of matured lamellae bone. All slices also showed healthy bone marrow with a regular number of hematopoietic cells and adipocytes. At low magnifications, there was no qualitative difference between PI and SPI.

Higher magnifications of the interface region of PI and SPI after 2 and 8 weeks are shown in Figure 6B–E. For both groups, the bone formation took place in the direction of the implant surface. Darker stained new bone could be seen next to the lighter stained old bone. Especially in the PI group, a soft tissue barrier of several micrometers between new bone and the implant surface occurred (Figure 6C,E). The implant surface in these regions was covered with a formation of elongated irregular-shaped giant cells. Other areas showed a one and two-layered stack of smaller and darker stained cells in close proximity to the implant surface. Further, towards the soft tissue, lighter stained cells were visible, among other fibroblasts.

Conversely, more bone-forming regions with direct bone contact could be observed for implants of the SPI group (Figure 6B,D). A pronounced portion of non-mineralized tissue was evident around the implant after 2 weeks (Figure 6B). It appeared as osteoid with included osteocytes in contact with the implant surface. Therefore, osteogenesis occurred in the SPI group in both directions, from the old bone, and from the implant surface. However, osteoblastic rims at osteoid formation sides were difficult to detect. After 8 weeks, the osteoid at the implant surface has mineralized and matured into the lamellar bone with blood vessels in the vicinity of the implant. Osteocytes arranged parallel to the surface showed a vital contact to the implant through cellular extensions.

### 3.7. Implant Surface Characterization

**Figure 7:**
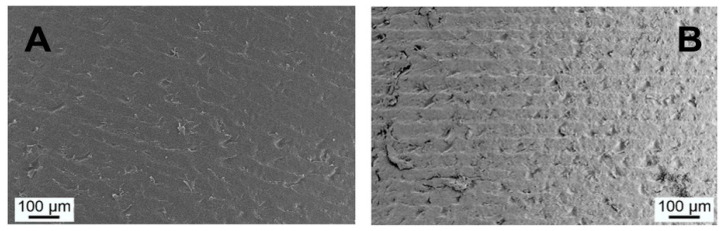
SEM images of the unmodified implant surface (PI) (**A**) post-production and (**B**) after 8 weeks in vivo. Almost no tissue remains on the surface after pull-out test.

After finishing the biomechanical investigation, pulled-out implants (PI and SPI) were supercritically dried for electron microscopic characterization of the interface. SEM analyses confirmed that all implants were removed almost without any residual tissue at the surface. When comparing the implant surface topography of the PI group before implantation and after the in vivo study, no specific change can be seen. This situation is representatively pictured in the SEM image Figure 7A for implant post-production and after 8 weeks in vivo, Figure 7B. Neither bone nor coverage with soft tissue was found at the implant surface. These results are in accordance with EDX investigations, which confirm the absence of calcium and phosphor as elements occurring in the inorganic portion of bone.

**Figure 8:**
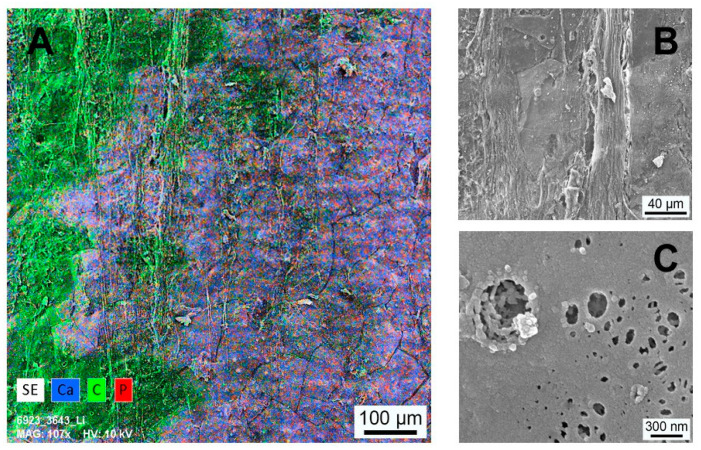
(**A**) EDX mapping of the implant surface (SPI) 8 weeks postoperative. The positions of the elements calcium, carbon and phosphor are indicated in blue, green and red, respectively. Areas covered with soft tissue are correlated with carbon. The residual surface shows higher concentration of calcium and phosphor. (**B**) Higher magnification of a surface portion with fibrous soft tissue coverage. (**C**) Smooth extracellular matrix covering areas with Ca and P occurrence—irregularities in this matrix exhibit an underlying nanostructure.

In contrast to the PI group, the surface of the SPI implants showed an altered surface topography, pictured in the SEM image Figure 8A for an implant after 8 weeks in vivo. Regardless of the time point, the surface was covered with remains of soft tissue. A qualitative analysis of the surface composition is pictured for the elements calcium, carbon and phosphor as EDX-mapping in Figure 8A. Areas with soft tissue were mainly composed of carbon without the bone-specific elements calcium and phosphor. In contrast, these elements were abundant in regions without soft tissue. These findings applied over the whole test period until 8 weeks.

Higher magnifications of areas with tissue coverage and without are pictured in Figure 8A,C, respectively. The structure of the detected regions with soft tissue was mainly governed by fibrous tissue. Regions without fibrous tissue were covered with a smooth extracellular matrix. Irregularities in the coverage of this matrix exposed an underlying structure with agglomerates of plate-like particles in the nanometer range.

## 4. Discussion

Pure HA, with its strong bond to native bone, is supposed to alter the bioinert surface of PEEK in a more favorable way regarding osseointegration [13,30,31]. However, a pure composite material will change the tensile strength of the bulk in an unfavorable way. Therefore, the aim of the study was to create a bioactive surface modification on PEEK implants, investigating the influence of its properties after 2, 4 and 8 weeks in a femur rat animal model. The unique coating is a compound of a pure biomaterial layer with an underlying interfacial nanocomposite layer based on the biomaterial and PEEK. As a transition zone between coating and implant, the interface is supposed to firmly connect the coating material with the implant surface. Moreover, the interface is hypothesized to prevent the complete remodeling of the biomaterial in it to enhance sustainable bone conductive properties of the implant surface.

Histological and histomorphometrical results of the study at hand demonstrate that the coated implants with interfacial composite layer (SPI) sustainably improve osseointegration compared to the uncoated pure PEEK implants (PI). The observed mean BIC of the SiO_2_/HA-coated PEEK implants after eight weeks is comparable to the earlier reported BIC values of PEEK implants with a plasma-sprayed titanium coating [32]. Similar to titanium surfaces, which are known for high osteoprogenitor cell adhesions [33], HA surfaces are also favorable for the adhesion of osteoprogenitor cells [34]. While at the moment, high numbers of osteoprogenitor cells are only proven for titanium-coated PEEK implant surfaces, the noted HA surface osteoprogenitor cell connection might be the reason for the improved BIC values in the presented study with SiO_2_/HA-coated PEEK implants. Generally, these findings support the known approach of adding HA and HA-based biomaterials to PEEK surfaces for better osseointegration [13,35,36]. In contrast to studies focusing on surface modifications but lacking pull-out or push-out tests [36,37], in the presented study, the fixed bone–implant connection between the coated PEEK implants and the surrounding bone is underlined by the two-times higher pull-out forces needed in SPI. Taking the pure cylindrical shape of the used implants in mind, factors such as the screw design of implants do not have to be considered as additional interfering factors. Therefore, the higher pull-out forces can be seen as direct proof of the increased bone–implant connection and underpin the improved osseointegration of the coated implants.

The applied coating consists of HA nanocrystals being morphologically identical to biological HA [38], which are embedded in a silica gel matrix. Due to the wet chemical route, the resulting silica gel is a weakly cross-linked network characterized by numerous open bonds (Si–O^−^ and Si–OH groups) and high porosity. The composition of HA and SiO_2_ forms a matrix with interconnecting nanopores generating a high surface area (Figure 3A). Since the structural properties of the coating material and the original bone graft are based on the same technology, results concerning the biological degradation and the effects on osteogenesis are transferable. Several in vivo studies have shown high biocompatibility of the fully degradable synthetic bone graft in different shapes (e.g., as granulate or microspheres) and a fast formation of new bone [39]. The early substitution of the silica matrix within view days by autologous molecules and bone-specific proteins with known functions in attraction, adhesion and differentiation of bone cells were regarded as the key mechanism of the bone graft’s function [40,41]. Despite this autologous self-coating process, the released silica is in general known to play a biochemical role in bone formation and mineralization [42,43]. Adam et al., who coated titan implants with a similar nanostructured SiO_2_/HA combination, observed in vitro a drastic decrease in the silica within the coating material shortly after immersion of the coated implants in human blood [24]. This behavior seems similar to the earlier described exchange in the silica gel matrix with an autologous extracellular matrix in the pure nanostructured SiO_2_/HA bone substitute material [41]. In the presented in vivo study, this known mechanism of the SiO_2_ within the coating material might additionally induce a considerable contribution to the enhanced BIC and generally direct bone formation on the implant surface.

Typically, temporary coatings or damages in the coating’s integrity expose the underlying implant surface. In the case of the PEEK surface, the exposure will lead to a non-connection between bone and implant material and might also decrease bone to implant contact in the long run. In the study at hand, even after 8 weeks, SEM analyses still revealed fractions of calcium, carbon and phosphor in SPI, while in the group of the pure PEEK implants (PI), an absence of calcium and phosphor was found. This finding, in combination with the observed underlying nanostructure (Figure 8B), might be caused by the interfacial composite layer. Osteoclast with their known size in the micrometer range is three magnitudes bigger than the primary pore size in the coating material, which might delay or even prevent the ability of osteoclasts to degrade the SiO_2_/HA-based coating within the nanocomposite layer. In the end, the applied coating leads to both a chemical and structural modification of the PEEK implant surface that enabled a vital, long-lasting connection between the modified PEEK implant surface and the surrounding bone. The promising results on the interface support the hypothesis that the combination of the nanostructured surface and the biochemical imitation of organic and inorganic components of bone [44] in the interface increases the direct apposition of bone and its vital bond with the implant surface.

Focusing on the question of long-term stability of the used implant surface modification, increased BIC and pull-out force values were found still in SPI (coated implants) after 8 weeks. Based on the observation time to rat lifetime ratio in the experiment, the observed data suggest sustainable stability of the nanostructured SiO_2_/HA coating modification. In relation to the human-to-rat age ratio [45], our results theoretically cover even the lifespan of dental implants in humans.

In conclusion, the HA/SiO_2_-based bioactive coating with interfacial composite on PEEK implants created a lasting bone-implant interface and might be a promising way to alter the bioinert surface property of PEEK-based implants permanently. Despite the presented results in the study at hand, further research regarding the underlying chemical and physical structure of the interfacial composite layer in vivo is needed. Particularly the exact mechanisms on cellular levels enabling the connection between the SiO_2_/HA coating and the surrounding bone tissue are unknown and should be subject to future research. Towards clinical applications, more complex implants modified with the nanostructured SiO_2_/HA need to prove the implant stability in vivo.

## 5. Patents

The coating process described in the study at hand is connected with the US Patent for “Osteoconductive coating of implants made of plastic” (Patent # 9,833,319).

## Data Availability

Data is contained within the article.
